# The PEP-pyruvate-oxaloacetate node: variation at the heart of metabolism

**DOI:** 10.1093/femsre/fuaa061

**Published:** 2020-12-08

**Authors:** Jeroen G Koendjbiharie, Richard van Kranenburg, Servé W M Kengen

**Affiliations:** Laboratory of Microbiology, Wageningen University, Stippeneng4, 6708 WE Wageningen, The Netherlands; Laboratory of Microbiology, Wageningen University, Stippeneng4, 6708 WE Wageningen, The Netherlands; Corbion, Arkelsedijk 46, 4206 AC Gorinchem, The Netherlands; Laboratory of Microbiology, Wageningen University, Stippeneng4, 6708 WE Wageningen, The Netherlands

**Keywords:** phosphoenolpyruvate, pyruvate, oxaloacetate, PPO-node, central metabolism, enzyme biochemistry

## Abstract

At the junction between the glycolysis and the tricarboxylic acid cycle—as well as various other metabolic pathways—lies the phosphoenolpyruvate (PEP)-pyruvate-oxaloacetate node (PPO-node). These three metabolites form the core of a network involving at least eleven different types of enzymes, each with numerous subtypes. Obviously, no single organism maintains each of these eleven enzymes; instead, different organisms possess different subsets in their PPO-node, which results in a remarkable degree of variation, despite connecting such deeply conserved metabolic pathways as the glycolysis and the tricarboxylic acid cycle. The PPO-node enzymes play a crucial role in cellular energetics, with most of them involved in (de)phosphorylation of nucleotide phosphates, while those responsible for malate conversion are important redox enzymes. Variations in PPO-node therefore reflect the different energetic niches that organisms can occupy. In this review, we give an overview of the biochemistry of these eleven PPO-node enzymes. We attempt to highlight the variation that exists, both in PPO-node compositions, as well as in the roles that the enzymes can have within those different settings, through various recent discoveries in both bacteria and archaea that reveal deviations from canonical functions.

## Teaser

Three metabolites, phosphoenolpyruvate, pyruvate and oxaloacetate together form the PPO-node, and are interconnected by a subset of 11 different enzymes; the current knowledge regarding these enzymes at the heart of metabolism is presented.

## INTRODUCTION

**Figure 1.**
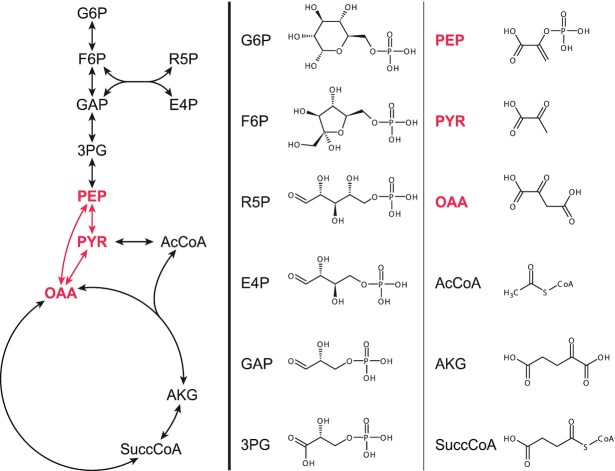
**Structural formulas of the 12 universal precursor metabolites and the topology of the core metabolism that connects them**. The PPO-node is highlighted in red. G6P, d-glucose-6-phosphate; F6P, d-fructose-6-phosphate; R5P, d-ribose-5-phosphate; E4P, d-erythrose-4-phosphate; GAP, d-glyceraldehyde-3-phosphate; 3PG, glycerate-3-phosphate; PEP, phosphoenolpyruvate; PYR, pyruvate; OAA, oxaloacetate; AcCoA, acetyl-CoA; AKG, α-ketoglutarate; SuccCoA, succinyl-CoA.

Different organisms can have many differences in their metabolism, yet they universally share twelve precursor metabolites that form the basis of all biomass on earth (Fig. 1) (Noor *et al*. 2010). Although significant variation exists, the set of reactions connecting those twelve precursor metabolites are generally well conserved and will be referred to as the *core metabolism*. The majority of the twelve precursor metabolites are part of the glycolysis, which is a largely linear sequence of reactions. The three precursor metabolites at the end of the glycolysis, phosphoenolpyruvate (PEP), pyruvate and oxaloacetate form a node with often multiple reactions connecting them. This PEP-pyruvate-oxaloacetate node (PPO-node) lies at the junction between the glycolysis and the TCA cycle, as well as many other metabolic pathways, and as such lies at the heart of the metabolism.

PEP has the highest-energy phosphate bond of all known natural organo-phosphates (Bowman *et al*. 1988), and serves as a precursor for aromatic amino acids (Herrmann and Weaver 1999). Pyruvate is a precursor for alanine, valine, leucine, isoleucine and lysine,^1^ and is also important as the first entry point for many fermentation pathways that re-oxidize NAD(P)H (Müller 2008). Oxaloacetate is part of the tricarboxylic acid cycle, where it accepts acetyl-CoA or is reduced to malate. Oxaloacetate also functions as a precursor for aspartate, which in turn is used for the biosynthesis of many other amino acids, as well as nucleotides (Park and Lee 2010).

**Figure 2.**
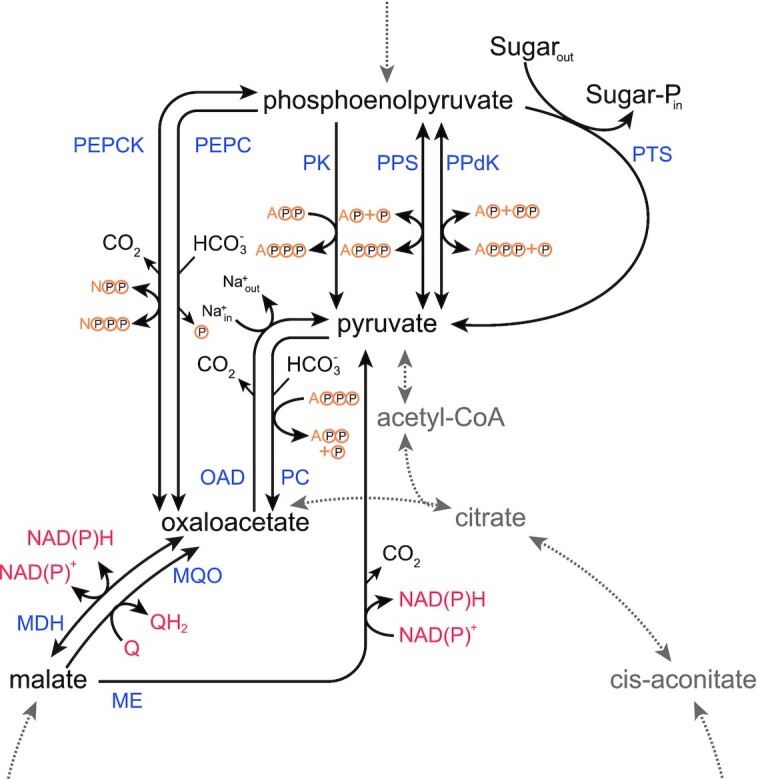
**Overview of all known reactions in the PPO-node**. MDH: Malate dehydrogenase, ME: Malic enzyme, MQO: Malate:quinone oxidoreductase, OAD: Oxaloacetate decarboxylase, PC: Pyruvate carboxylase, PEPC: Phosphoenolpyruvate carboxylase, PEPCK: Phosphoenolpyruvate carboxykinase, PK: Pyruvate kinase, PPdK: Pyruvate phosphate dikinase, PPS: Phosphoenolpyruvate synthase, PTS: Phosphotransferase system. The yellow circles represent phosphate (groups).

Yet, despite forming the heart of metabolism, the PPO-node is also the part of the core metabolism that seems to vary the most between different organisms, as many possible biochemical reactions are known to exist in the PPO-node, summarized in Fig. 2. This is in stark contrast with the lower part, or trunk of glycolysis, directly leading towards the PPO-node (starting with glyceraldehyde-3-phosphate), which is one of the oldest and most conserved metabolic pathways that exist^2^ (Ronimus and Morgan 2003).

The conversion of PEP to pyruvate—typically considered the last step of the glycolysis—is also the step where the glycolytic energy is harvested, in the form of ATP (equivalents). This conversion can either occur directly, through pyruvate kinase, pyruvate phosphate dikinase,^3^ the phosphotransferase system, or even phosphoenolpyruvate synthase; or can occur via the intermediate formation of oxaloacetate, with or without malate as intermediate as well (Fig. 2). And not only do each of these different routes have different enzymes associated with (with different properties), each route also has a different set of cofactors that are used (i.e. red and yellow elements in Fig. 2). Variations in the PPO-node are therefore profoundly linked to cellular energetics and the constraints on them.

Anaerobic and extremophilic microorganisms most often show deviations from the ‘typical’ central metabolism found in most model organisms, as a consequence of the way in which the different constraints that dictate cellular metabolism are prioritized (differently) in ‘extreme’ environments.^4^ Research into the PPO-node enzymes of anaerobic- and extremophilic bacteria and archaea will therefore be featured extensively in this review, where we aim to give a concise view of what is currently known about the biochemical reactions that form the PPO-node, putting emphasis on the variations that exist.

## THE DIFFERENT ENZYMES OF THE PPO-NODE^5^

### Pyruvate kinase (EC 2.7.1.40)

$$\begin{eqnarray*}
{\rm{Phosphoenolpyruvate}} + {\rm{ADP}} \to {\rm{Pyruvate}} + {\rm{ATP}}\nonumber \\
\quad {\Delta _r}{{\rm{G'}}^{\rm{m}}} = - 27.7 \pm 0.8{\rm{ kJ}}/{\rm{mol}}
\end{eqnarray*}$$

Pyruvate kinase (PK) catalyzes the transphosphorylation from phosphoenolpyruvate (PEP) to ADP, forming pyruvate and ATP, which is typically the final, and a rate determining step of the glycolysis. The reaction proceeds in two steps. First, the phosphoryl-group is transferred from PEP to ADP, and then the enolate that is formed is converted into pyruvate via the addition of a proton (Kumar and Barth 2010). It has to be noted, however, that the specificity for ADP is very low, as many other NDPs can function as an acceptor (Weber 1969; Abbe and Yamada 1982; Sakai, Suzuki and Imahori3 1986). The reaction is typically considered irreversible, however, recent findings suggest that the Δ_r_G of PK is not as negative as was previously thought (Park *et al*. 2016). PK has an absolute requirement for divalent cations, in particular Mg^2+^ (Enriqueta Muñoz and Ponce 2003). Phylogenetically, PKs can be clustered in two distinct groups, K^+^-dependent (cluster I) and K^+^-independent (cluster II) enzymes. These two clusters do not correlate with the three domains of life (Schramm *et al*. 2000; Johnsen, Hansen and Schönheit 2003; Oria-Hernández, Riveros-Rosas and Ramírez-Sílva 2006; Guerrero-Mendiola *et al*. 2017), although PKs are ubiquitously present in all three, with only a few exceptions that will be discussed below (Enriqueta Muñoz and Ponce 2003). Members of the gammaproteobacteria, including *Escherichia coli*, *Yersinia enterocolitica* and *Salmonella typhimurium*, are unique in that they possess isozymes of both the K^+^-dependent and the K^+^-independent clusters (Guerrero-Mendiola *et al*. 2017). For *Y. enterocolitica* it was shown that both PykF (cluster I) and PykA (cluster II) form homotetramers, which is the most common quaternary structure for PKs (Enriqueta Muñoz and Ponce 2003), although monomers (Knowles, Dennis and Plaxton 1989), homodimers (Pawluk, Scopes and Griffiths-Smith 1986), heterotetramers (Plaxton 1989), heterohexamers (Plaxton, Smith and Knowles 2002), and homodecamers (Lin, Turpin and Plaxton 1989) are also known to exist.

Pyruvate kinase is responsible for one of the three rate determining steps, and one of the two ATP-generating steps of glycolysis. Consequently, its regulation is complex and features many layers; especially its allosteric regulation is exceptionally intricate, and is different between the two phylogenetic clusters (Valentini *et al*. 2000). K^+^-dependent PKs are stimulated by bi-phosphorylated sugars, such as fructose-1,6-bisphosphate (Jurica *et al*. 1998) or fructose-2,6-bisphosphate (Schaftingen, Opperdoes and Hers 1985; Ernest *et al*. 1994), whereas K^+^-independent PKs are generally allosterically regulated by AMP and mono-phosphorylated sugars, such as glucose-6-phosphate and ribose-5-phosphate (Yamada and Carlsson 1975; Malcovati and Valentini 1982; Guerrero-Mendiola *et al*. 2017). Several PKs have been described to deviate from this typical allosteric control. The PK from the hyperthermophilic archaeon *Pyrobaculum aerophilum* was found to respond to 3-phosphoglycerate, rather than to any of the previously mentioned effectors (Solomons *et al*. 2013), and the PKs from the parasite *Cryptosporidium parvum* and other hyperthermophilic archaea have no known effectors at all (Denton *et al*. 1996; Schramm *et al*. 2000; Johnsen, Hansen and Schönheit 2003; Cook *et al*. 2012). In *Corynebacterium glutamicum*, PK is subject to serine-phosphorylation. Whether the PK activity is controlled via this post-translational modification is not known (Bendt *et al*. 2003).

Besides the kinase activity, PKs also have an intrinsic and conserved oxaloacetate-decarboxylase activity. And although this decarboxylase activity is rather low and likely the result of a non-mutable requirement in the active site, a potential biological role cannot be ruled out (Zhong *et al*. 2014).

**Figure 3.**
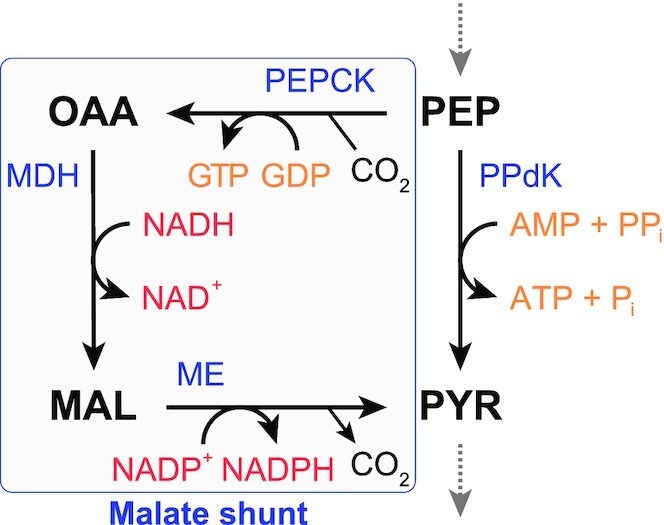
**Conversion of PEP to pyruvate through the malate shunt in the PPO-node of C. thermocellum**. The malate shunt results in the transhydrogenation of NADH to NADPH. MAL: malate, MDH: malate dehydrogenase, ME: malic enzume, OAA: oxaloacetate, PEP: phosphoenolpyruvate, PEPCK: PEP carboxykinase, PPdK: pyruvate phosphate dikinase, PYR: pyruvate.

As mentioned, only a few (sugar fermenting) organisms are known that do not possess a PK. These include fermentative protists, such as *Entamoeba histolytica* and *Tritrichomonas foetus* (Hrdý, Mertens and Van Schaftingen 1993; Mertens 1993; Saavedra-Lira, Ramirez-Silva and Perez-Montfort 1998), and the thermophilic bacterium *Clostridium thermocellum*, the best cellulose degrader that is currently known (Zhou *et al*. 2013; Olson *et al*. 2016). For the latter, it was recently proven that conversion of PEP to pyruvate proceeds via the ‘malate shunt’ and via pyruvate phosphate dikinase, as shown in Fig. 3 (Olson *et al*. 2016). The malate shunt, running via PEP carboxykinase, malate dehydrogenase, and malic enzyme accounts for a third of the flux to pyruvate, with the remainder going via pyruvate phosphate dikinase. Interestingly, the malate shunt could only be disrupted after introduction of a heterologous PK (Deng *et al*. 2013), whereas pyruvate phosphate dikinase could be knocked-out in the wild-type strain, redirecting all flux via the malate shunt (Olson *et al*. 2016). In fermentative protists, the function of PK is replaced by pyruvate phosphate dikinase as well (Saavedra *et al*. 2019). Flux through the malate shunt has not been described in those cases, but it is possible that this pathway also plays a role in fermentative protists. Overall, the malate shunt is an efficient mechanism for the transhydrogenation of NADH to NADPH, considering that NADPH generation through the pentose-phosphate pathway results in loss of substrate in the form of CO_2_ and that (bifurcating) transhydrogenases require high-energy electron donors (i.e. ferredoxin or flavodoxin) (Wang *et al*. 2010).

Almost paradoxically, in *C. glutamicum* PK was also found to be important for growth on certain gluconeogenic substrates, including acetate and citrate, which are first converted to PEP via PEP carboxykinase (Netzer *et al*. 2004). PK is then required for the formation of pyruvate-derived biomass constituents.

### Pyruvate phosphate dikinase (EC 2.7.9.1)

$$\begin{equation*}
{\rm{Pyruvate}} + {\rm{ATP}} + {{\rm{P}}_{\rm{i}}} \leftrightarrow {\rm{Phosphoenolpyruvate}} + {\rm{AMP}} + {\rm{P}}{{\rm{P}}_{\rm{i}}} + {{\rm{H}}^ {+} }\nonumber \\
\quad {\Delta _{\rm{r}}}{{\rm{G^{\prime}}}^{\rm{m}}} = 19.6 \pm 1.0{\rm{ kJ}}/{\rm{mol}}
\end{equation*}$$

Pyruvate phosphate dikinase (PPdK) catalyzes the reversible conversion of PEP to pyruvate via three partial reactions: First, PPdK combines with ATP to form AMP and the enzyme-diphosphate, then pyrophosphate is formed via the combination of orthophosphate and a phosphate group from the enzyme-diphosphate. Finally, the other phosphate bound to PPdK is transferred to pyruvate, forming PEP (Evans and Wood 1968). The mechanism involves one of the largest single protein-domain movements known so far (Minges, Höppner and Groth 2017), and is believed to proceed via an alternate binding change mechanism, akin to ATP synthase (Ciupka and Gohlke 2017).

Most of what we know about PPdK comes from studies with plants and parasitic protists, but PPdKs are also present in archaea and in bacteria, from which they are in fact believed to originate (Slamovits and Keeling 2006). In C4 plants, PPdK is responsible for the first step of carbon-fixation starting with pyruvate, and can comprise up to 10% of soluble protein in C4 leaves (Chastain 2010). It is no surprise then that PPdK has mostly been studied in plants, and is therefore typically considered a gluconeogenic enzyme, which actually is surprising, in view of the Δ_r_G′^m^. Furthermore, for several bacteria, including *Microbispora rosea* and *Acetobacter xylinum* PPdK was shown to be important during gluconeogenesis as well (Benziman and Eizen 1971; Eisaki *et al*. 1999). However, the thermodynamics certainly do not restrict it from working in the glycolytic direction. In fact, for a multitude of protists (Deramchia *et al*. 2014; González-Marcano *et al*. 2016; Rivero *et al*. 2016) and bacteria (Reeves 1968; Bielen *et al*. 2010; Olson *et al*. 2016), and the archaeon *Thermoproteus tenax* (Tjaden *et al*. 2006), PPdK has been demonstrated to function as a glycolytic enzyme, which is likely also its original function. In C3 plants, PPdK is ubiquitously present in the plant tissue, where its role is currently not well understood, since those plants do not use PPdK for carbon fixation (Chastain *et al*. 2011; Hyskova and Ryslava 2016).

In bacteria (Ernst, Budde and Chollet 1986; Ciupka and Gohlke 2017) and archaea (Tjaden *et al*. 2006), PPdK appears to be active in homodimeric form, whereas for C4 plants it typically functions optimally as a homotetramer (Shirahashi, Hayakawa and Sugiyama 1978; Ohta, Ishida and Usami 2004). For protists, both dimeric (Hiltpold, Thomas and Köhler 1999) and tetrameric (Saavedra-Lira, Ramirez-Silva and Perez-Montfort 1998) forms have been described. Free Mg^2+^ is required for the formation of PPdK tetramers in plants and thus its activity, whereas inactivation occurs at low temperatures due to dissociation into dimers and monomers. For the dimeric PPdK, neither Mg^2+^ nor low temperature affects the stability of the quaternary structure, but Mg^2+^ is still required for its catalytic activity (Hiltpold, Thomas and Köhler 1999). Other effectors described to stimulate PPdK activity in plants, protists and bacteria are the monovalent cations NH_4_^+^ and K^+^, whereas PPdKs are not subject to regulation by metabolites. Furthermore, pH significantly influences the direction that is favoured, with low pH favouring the glycolytic (proton consuming) direction, and vice versa (Chastain 2010). The PPdK of the archaeon *T. tenax*, on the contrary, is not affected by any monovalent cations. Instead, ATP was found to act as an potent competitive inhibitor with AMP (Tjaden *et al*. 2006).

In C4 plants, PPdK activity is tightly regulated via inactivation and activation by the PPdK regulatory protein (PDRP), respectively through ADP-dependent phosphorylation and Pi-dependent dephosphorylation of a threonine residue in the active site. This regulation is imposed by light intensity, and is believed to be dictated by the adenylate energy charge, which is low under dark conditions (Edwards *et al*. 1985; Chastain 2010). A homolog of the PDRP (DUF 299) is also present in all bacteria harbouring PPdK, and was shown in *Listeria monocytogenes* to activate and inactivate PPdK via an identical mechanism as described for plant PPdK (Tolentino, Chastain and Burnell 2013). Bacteria harbouring PDRP without PPdK also exist, in which case PDRP likely controls the activity of PEP synthase, as was shown in *E. coli* (Burnell 2010). The DUF299 protein is absent in archaea and protists (Burnell 2010; Chastain *et al*. 2011), although inactivation via phosphorylation of the threonine residue still occurs in the PPdK from *Trypanosoma cruzi*. Furthermore, *T. cruzi* PPdK is irreversibly inactivated via proteolytic cleavage from 100 to 75 kDa, after which the protein associates with the glycosomal membrane (González-Marcano *et al*. 2014). Nevertheless, the full extent of the regulation via posttranslational modification of PPdK remains unknown, as multiple phosphorylation sites have been discovered more recently in plants, of which not all are phosphorylated by PDRP, as well as an acetylation site near the N-terminus, whose function is unknown (Chen *et al*. 2014).

### Phosphoenolpyruvate synthase (EC 2.7.9.2)

$$\begin{eqnarray*}
{\rm{Pyruvate}} + {\rm{ATP}} \leftrightarrow {\rm{Phosphoenolpyruvate}} + {\rm{AMP}} + {{\rm{P}}_{\rm{i}}}\nonumber \\
\quad {\Delta _{\rm{r}}}{{\rm{G'}}^{\rm{m}}} = - 13.3 \pm 1.0{\rm{ kJ}}/{\rm{mol}}
\end{eqnarray*}$$

**Figure 4.**
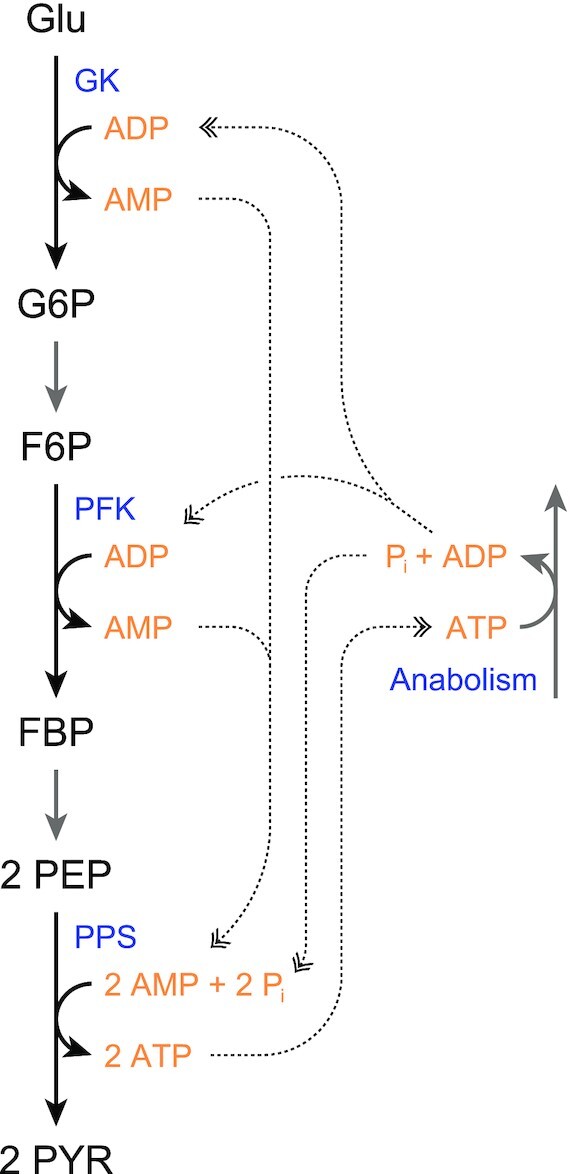
**Cycling of ATP/ADP/AMP in the metabolism of *T. kodakarensis*, which relies on PPS for glycolysis, as well as ADP-dependent GK and PFK**. F6P, fructose 6-phosphate; FBP, fructose 1,6-bisphosphate; G6P, glucose 6-phosphate; GK, glucokinase; Glu, glucose; PEP, phosphoenolpyruvate; PPS, PEP synthase; PFK, 6-phosphofructokinase; PYR, pyruvate.

Phosphoenolpyruvate synthase (PPS), or sometimes called pyruvate, water dikinase resembles pyruvate phosphate dikinase, both catalytically and structurally (Nguyen and Saier 1995). Like pyruvate phosphate dikinase, the reaction proceeds via three partial reactions, where first the enzyme-diphosphate is formed. The first phosphate group is then transferred to water, rather than orthophosphate, expending a high-energy phosphate bond. Finally, the second phosphate is transferred to pyruvate (Cooper and Kornberg 1967), forming PEP. As a consequence of the energy that is dissipated at the cost of the high-energy phosphate bond, the reaction strongly favours the gluconeogenic direction, to PEP. Nonetheless, in the extreme case of *Thermococcus kodakarensis*, but probably also in related *Thermococcus/Pyrococcus* species, PPS is responsible for the glycolytic conversion of PEP to pyruvate (Imanaka *et al*. 2006). This makes sense in view of the fact that *Thermococcus/Pyrococcus* species have an ADP-dependent glucokinase and 6-phosphofructokinase (Kengen *et al*. 1994)—forming AMP. This supply of AMP fits well with ATP synthesis from AMP by PPS, as shown in Fig. 4.

PPSs are present in all three domains of life. In eukaryotes, however, the majority of PPSs are annotated in a specific class of fungi (leotiomyceta), without any functional evidence available. Structural data regarding PEP synthase is limited to one resolved crystal structure, from *Neisseria meningitidis*. In the hyperthermophilic archaeon *Staphylothermus marinus*, PPS forms a large homomultimeric complex, composed of 24 subunits, assembled in a rather unusual octahedral arrangement. For *Pyrococcus furiosus*, PPS was found to exist in a large and a smaller homomultimeric complex, of which the smaller octamer was found to be the active form (Hutchins, Holden and Adams 2001). PPS from *T. tenax* also exists as a large homomultimer (Tjaden *et al*. 2006), so it is likely a general feature of PPSs from hyperthermophilic archaea, as PPS from *E. coli* is active as a homodimer (Narindrasorasak and Bridger 1977). Unfortunately, no other PPSs have been characterized in such detail.

All characterized PPSs require divalent cations for activity. Furthermore, *P. furiosus* PPS contains one Ca-atom per subunit, and its activity is stimulated by K^+^ and NH4^+^ (Hutchins, Holden and Adams 2001). The optimal pH of the PEP-forming reaction was 9.0, while the optimal pH of the pyruvate formation reaction was 7.5 (Hutchins, Holden and Adams 2001). Contrary to PPS from *P. furiosus*, and also *Methanothermobacter thermoautotrophicus*, activity of PPS from *T. tenax* is not stimulated by the monovalent cations (Tjaden *et al*. 2006). *T. tenax* PPS is strongly inhibited by 2-oxoglutarate, and AMP and ADP, competitively with pyruvate and ATP, respectively, in the PEP-forming direction (Tjaden *et al*. 2006). For *E. coli* PPS, Mg^2+^ and Mn^2+^ were required for activation, but higher concentrations led to decreased activity (Berman and Cohn 1970). As was already mentioned, it was shown that PPdK regulatory protein (PDRP) is a regulator for PPS activity in *E. coli*, analogous to its regulation of pyruvate phosphate dikinase (Burnell 2010). Nothing is known regarding post-translational modification in archaeal PPSs.

In *T. tenax*, PPS is downregulated during heterotrophic growth and upregulated during autotrophic growth, indicative of a gluconeogenic role (Tjaden *et al*. 2006), whereas in *T. kodakarensis*, transcript levels of PPS increased under glycolytic conditions when compared with cells grown on pyruvate or amino acids, indicating a glycolytic role, which was confirmed by the knock-out of PPS (Imanaka *et al*. 2006). *T. kodakarensis* also harbours a pyruvate kinase, but this appeared to be less essential for glycolysis than PPS (Imanaka *et al*. 2006). In line with this, PPS from *P. furiosus* is also considered to function in the pyruvate-forming direction, and was shown to be upregulated by maltose (Sakuraba et al. 1999, 2001; Sakuraba and Ohshima 2002). In most organisms, however, PPS functions as a gluconeogenic enzyme. Knock-outs in *E. coli* and *S. typhimurium* showed that it is essential for growth on C3 substrates, such as pyruvate (Smyer and Jeter 1989). In *M. thermoautotrophicus*, like for *T. tenax*, PPS plays a role in autotrophic growth (Eyzaguirre, Jansen and Fuchs 1982).

### Phosphoenolpyruvate carboxylase (EC 4.1.1.31)

$$\begin{eqnarray*}
{\rm{Phosphoenolpyruvate}} + {\rm{HC}}{{\rm{O}}_{3}}^ {-} \leftrightarrow {\rm{Oxaloacetate}} + {{\rm{P}}_{\rm{i}}}\nonumber \\
\quad {\Delta _{\rm{r}}}{{\rm{G^{\prime}}}^{\rm{m}}} = - 33.6 \pm 6.4{\rm{ kJ}}/{\rm{mol}}
\end{eqnarray*}$$

Phosphoenolpyruvate carboxylase (PEPC) catalyzes the irreversible bicarbonate fixation on PEP, yielding oxaloacetate and orthophosphate. This reaction likely proceeds via the following three steps: First, carboxyphosphate and enolate are formed by the transfer of the phosphate group. The carboxyphosphate is then cleaved into hydrogen phosphate and the—compared to bicarbonate—more reactive CO_2_. Finally, the CO_2_ is fixed on the enolate, forming oxaloacetate (Kai, Matsumura and Izui 2003). This reaction is highly exergonic, and essentially irreversible.

**Figure 5.**
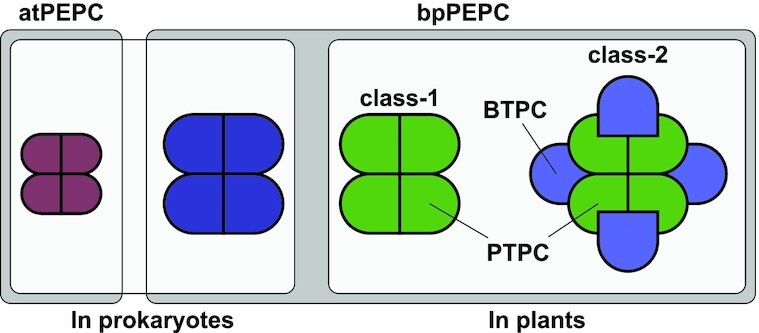
**Overview of the different PEPC complexes and the typically used nomenclature**. atPEPC, archaeal-type PEPCs; bpPEPC, bacterial and plant PEPC. Most PEPCs are tetrameric, except for the class-2 PEPCs in plants, which is a hetero-octameric combination of the plant-type PEPCs (PTPC; in green) and bacterial-type PEPC (BTPC; in light-blue). Although PTPC and BTPC are both bpPEPCs, BTPCs are more homologous to those found in Bacteria than the others found in plants (i.e. class-1/PTPC).

PEPC is present in plants, bacteria and archaea, but not in fungi and animals. The archaeal-type PEPC, which is also present is several bacteria, differs strongly from its counterpart found in plants and bacteria (Ettema *et al*. 2004; Patel, Kraszewski and Mukhopadhyay 2004). As with pyruvate phosphate dikinase, PEPC plays a crucial role in C4 photosynthesis, and consequently, a lot of what we know about PEPC comes from plants. Fortunately, significant effort has gone into investigating both bacterial and archaeal PEPCs as well. The most obvious difference between the PEPC from bacteria and plants (bpPEPC) compared to the archaeal-type PEPC (atPEPC), is the size; the atPEPC, with monomer sizes ranging from 55 to 60 kDa, is a lot smaller than the bpPEPC, which ranges from 90 to 110 kDa (Ettema *et al*. 2004). Nevertheless, bpPEPC and atPEPC both exist as homotetramers, with two sets of dimers being somewhat loosely associated with each other (Izui *et al*. 2004; Dharmarajan *et al*. 2011). In plants and algae, a second class of PEPCs has been discovered that forms a hetero-octameric complex, consisting of four typical ‘class-1’ plant-type monomers, and four monomers related to bacterial PEPC, typically referred to as bacterial-type PEPC (BTPC) to contrast them with the plant-type PEPCs (PTPC) (O'Leary *et al*. 2009). Thus, bpPEPCs encompass three separate phylogenetic groups, bacterial PEPCs, BTPCs, and PTPCs (O'Leary, Park and Plaxton 2011). Together with the atPEPCs, four main PEPC types can therefore be distinguished, summarized in Fig. 5.

Many different allosteric effectors for PEPC are known, and listing all known effectors, and the variations of allosteric control in different organisms is beyond the scope of this review. Generally, class-1 PEPCs in plants are inhibited by malate and activated by glucose-6-phosphate. Aspartate and glutamate are important feedback effectors in tissues active in nitrogen assimilation and/or transamination reactions (O'Leary, Park and Plaxton 2011). In bacteria, PEPC is typically activated by fructose 1,6-bisphosphate and acetyl-CoA, and inhibited by aspartate and malate (Chen *et al*. 2002). Individually, the effects of those allosteric regulators are rather modest. However, for *E. coli* it was shown that at physiological concentrations, the combination of these effectors turned fructose 1,6-bisphosphate into an ultrasensitive allosteric regulator for PEPC. Upon fructose 1,6-bisphosphate depletion resulting from glucose removal, PEPC is very rapidly virtually switched off, which is important for the build-up of PEP, required for future glucose import via the phosphotransferase system (Xu *et al*. 2012). In cyanobacteria and plants, the allosteric control is determined by a single amino acid residue (Takeya, Hirai and Osanai 2017). The hetero-octameric class-2 PEPCs from plants are found to have significantly lower sensitivity for allosteric control compared to class-1 PEPCs, which is believed to allow them to bypass PEPC inhibition under specific conditions that would inhibit class-1 PEPCs (O'Leary *et al*. 2009). It was also shown that class-2 PEPC expression correlates with the formation of storage organelles in pollen, so its function might also be related to the formation of storage molecules (Igawa *et al*. 2010). The atPEPCs have no known allosteric activators, and residues related to the binding of inhibitors in bpPEPCs are absent (Matsumura, Izui and Mizuguchi 2006; Dharmarajan *et al*. 2011). Inhibition of atPEPC by aspartate was found to be competitive with PEP, rather than allosteric (Dharmarajan *et al*. 2011).

In plants, PEPC functionality is also controlled via phosphorylation, by PEPC protein kinase, and via mono-ubiquitination (Aldous *et al*. 2014; Ruiz-Ballesta *et al*. 2016; Ying *et al*. 2017). In bacteria, much less is known regarding PEPC control via post-translational modification. Recently, however, it was shown that in *C. glutamicum*, (possibly non-enzymatic) PEPC acetylation of lysine-653 decreased PEPC activity, which was activated again via enzymatic deacetylation.

### Phosphoenolpyruvate carboxykinase (EC 4.1.1.49) (EC 4.1.1.32) (EC 4.1.1.38)

$$\begin{eqnarray*}
{\rm{Oxaloacetate}} + {\rm{ATP}} \leftrightarrow {\rm{Phosphoenolpyruvate}} + {\rm{ADP}} + {\rm{CO}}\nonumber \\
\quad {\Delta _{\rm{r}}}{{\rm{G^{\prime}}}^{\rm{m}}} = - 6.8 \pm 6.2{\rm{ kJ}}/{\rm{mol}}
\end{eqnarray*}$$

$$\begin{eqnarray*}
{\rm{Oxaloacetate}} + {\rm{GTP}} \leftrightarrow {\rm{Phosphoenolpyruvate}} + {\rm{GDP}} + {\rm{C}}{{\rm{O}}_2}\nonumber \\
\quad {\Delta _{\rm{r}}}{{\rm{G^{\prime}}}^{\rm{m}}} = - 4.3 \pm 6.7{\rm{ kJ}}/{\rm{mol}}
\end{eqnarray*}$$

$$\begin{eqnarray*}
{\rm{Oxaloacetate}} + {\rm{P}}{{\rm{P}}_{\rm{i}}} \leftrightarrow {\rm{Phosphoenolpyruvate}} + {{\rm{P}}_{\rm{i}}} + {\rm{C}}{{\rm{O}}_2}\nonumber \\
\quad {\Delta _{\rm{r}}}{{\rm{G^{\prime}}}^{\rm{m}}} = 3.9 \pm {\rm{ }}6.2{\rm{ kJ}}/{\rm{mol}}
\end{eqnarray*}$$

Phosphoenolpyruvate carboxykinase (PEPCK) catalyzes the reversible decarboxylation and phosphorylation between oxaloacetate and PEP. Enzymes exist that can use either ATP, GTP or PP_i_ as phosphate donor (EC 4.1.1.49, 4.1.1.32 and 4.1.1.38, respectively). The enzymes using PP_i_ are not evolutionary related to the enzymes using either ATP or GTP, which do share an evolutionary origin, despite having relatively low sequence homology (Aich and Delbaere 2007; Chiba *et al*. 2015). PP_i_-PEPCK will be discussed further down, and for now, PEPCK will refer to the ATP- and GTP-dependent enzymes, which are believed to have a conserved mechanism of catalysis, despite the low sequence identity (Matte *et al*. 1997).

In contrast to phosphoenolpyruvate carboxylase, PEPCK uses CO_2_ rather than HCO_3_^−^, and has a specific binding site for CO_2_, which is uncommon for (de)carboxylating enzymes (Cotelesage *et al*. 2007). PEPCK requires both Mg^2+^ and Mn^2+^ for maximal activity, where Mg^2+^ is associated with the nucleotide and Mn^2+^ with the active site of the enzyme (Goldie and Sanwal 1980; Machová *et al*. 2015). The reaction is believed to proceed in a stepwise manner, with oxaloacetate binding first to the Mn^2+^-enzyme complex followed by binding of the Mg^2+^-nucleotide complex. Catalysis then proceeds by closing of a Ω–loop lid-domain, during which a stabilized enolate intermediate is formed after decarboxylation of oxaloacetate, allowing for phosphoryl transfer from the nucleotide, rather than the energetically more favourable protonation (Carlson and Holyoak 2009; Cui *et al*. 2017).

PEPCK is present in all domains of life. Mammalian PEPCKs are all GTP-dependent, and all plant PEPCKs are ATP-dependent. Archaeal PEPCKs are mostly GTP-dependent, and bacterial and other eukaryotic PEPCKs can be both ATP- and GTP-dependent (Aich and Delbaere 2007). No clear explanation exists for the use of either ATP or GTP. Except for bacterial ATP-PEPCKs, which are monomeric, most other ATP-PEPCKs are multimeric, with two, four, or six subunits (Matte *et al*. 1997). Conversely, most known GTP-PEPCKs are monomeric, with the exception of the sole characterized archaeal GTP-PEPCK, which forms a homotetramer (Fukuda *et al*. 2004).

GTP-PEPCKs are not subject to allosteric control, but for ATP-PEPCKs some allosteric effectors are reported (Fukuda *et al*. 2004): In *E. coli*, Ca^2+^ was shown to act as an allosteric activator (Goldie and Sanwal 1980), and in C4 plants, PEPCK was shown to be inhibited by 3-phosphoglycerate, fructose-6-P, fructose-1,6-bisphosphate and dihydroxyacetone phosphate (Burnell 1986).

In most organisms, the primary function of PEPCK is believed to be gluconeogenesis, in order to convert pyruvate to PEP; first by pyruvate carboxylase, followed by PEPCK. In some C4 plants, PEPCK is used for the localized enrichment of CO_2_ in the bundle-sheath cells. In contrast to (most) eukaryotes, many prokaryotes possess PPS and/or PPdK for the conversion of pyruvate to PEP; PEPCK (or malic enzyme/oxaloacetate decarboxylase) is required for the growth on TCA-intermediates such as malate and succinate (Liao, Chao and Patnaik 1994). The reaction operates close to equilibrium, and there are also more and more examples where PEPCK is responsible for the conversion of PEP to oxaloacetate fixing CO_2_. These notably include natural succinic acid producers, which thus are able to couple succinate fermentation to the generation of ATP-equivalents, but also other capnophilic (CO_2_-loving) bacteria (Laivenieks, Vieille and Zeikus 1997; Schöcke and Weimer 1997; Koendjbiharie, Wiersma and van Kranenburg 2018). In *C. thermocellum*—not a succinate producer—PEPCK, followed by malate dehydrogenase and malic enzyme, was shown to be involved in the glycolytic conversion of PEP to pyruvate, via the malate shunt (Olson *et al*. 2016). In *M. tuberculosis*, Fe^2+^ inhibits the gluconeogenic direction of PEPCK while it activates the anaplerotic oxaloacetate synthesis (Machová *et al*. 2015). The human cytosolic PEPCK—considered a gluconeogenic or cataplerotic enzyme (Yang, Kalhan and Hanson 2009)—was recently shown to be converted into an anaplerotic enzyme upon p300-dependent acetylation at high-glucose conditions (Latorre-Muro *et al*. 2018). Other post-translational control of cytosolic PEPCK in humans include phosphorylation, and ubiquitination.

PP_i_-PEPCK (or PEP carboxytransphosphorylase; PEPCT) is distributed in limited but diverse lineages of unicellular eukaryotes and bacteria, but not in archaea. Most of these organisms also possess the canonical ATP/GTP-PEPCKs; *E. histolytica*, however, possesses three PP_i_-PEPCK genes, but lacks any other PEPCK-types (Chiba *et al*. 2015; Saavedra *et al*. 2019). In *Propionibacterium shermanii*, PP_i_-PEPCK was found to require Mg^2+^, Mn^2+^, Co^2+^ for activity (Lochmüller, Wood and Davis 1966). Sulfate, malate and PP_i_ were found to be effective inhibitors.

### Pyruvate carboxylase (EC 6.4.1.1)

$$\begin{eqnarray*}
{\rm{Pyruvate}} + {\rm{HC}}{{\rm{O}}_3}^ {-} + {\rm{ATP}} \leftrightarrow {\rm{Oxaloacetate}} + {\rm{ADP}} + {{\rm{P}}_{\rm{i}}}\nonumber \\
\quad {\Delta _{\rm{r}}}{{\rm{G^{\prime}}}^{\rm{m}}} = - 5.9 \pm 6.4{\rm{ kJ}}/{\rm{mol}}
\end{eqnarray*}$$

Pyruvate carboxylase (PC) catalyzes the irreversible, ATP-dependent carboxylation of pyruvate by HCO_3_^−^ to oxaloacetate. The enzyme is a biotin-carboxylase, a family of enzymes that includes amongst others, acetyl-CoA carboxylase and urea carboxylase. Biotin-carboxylases contain three main functional domains: The biotin carboxylase (BC) responsible for the ATP-dependent carboxylation of the biotin with HCO_3_^−^; the biotin carboxyl carrier protein (BCCP) that has the biotin covalently linked to it and is responsible for the translocation of the carboxylated biotin to the third domain; and the carboxyltransferase (CT), where the CO_2_ is transferred to the acceptor, which is pyruvate in the case of PC. The BC and BCCP domains are conserved between biotin-carboxylases, whereas the CT domain is different, depending on the substrate.

Two different PC variants are known: The single-polypeptide enzyme and the two-subunit enzyme, in which the BC domain composes the α-chain, and the BCCP and CT domains the β-chain. The former is present in eukaryotes and bacteria and is only active as a tetramer (α_4_). The two-subunit enzyme is present in archaea and some bacteria and is active as a tetramer of the two subunits (α_4_β_4_) (Lai *et al*. 2006; Tong 2013). PC has an absolute requirement for one divalent cation bound per subunit, typically in the form of Mg^2+^, although isoforms requiring Zn^2+^ are also described (Jitrapakdee and Wallace 1999; St Maurice *et al*. 2007). Furthermore, K^+^ and larger monovalent cations can stimulate PC activity (Charles and Willer 1984; Scrutton and Taylor 1974). The tetramer is arranged as a dimer of dimers, where the BCCP domains swings between any of the two BC and two CT domains of a dimer, allowing four different translocation routes to take place (Liu *et al*. 2018).

The α_4_-type PC is allosterically activated by binding of acetyl-CoA to the BC domain, which specifically activates the translocation of BCCP from the BC domain of its own subunit to the CT domain of the opposing subunit. The α_4_β_4_-type PC is insensitive to acetyl-CoA. The degree of activation by acetyl-CoA can vary significantly, depending on the origin (Adina-Zada *et al*. 2012). A notable exception is the α_4_-type PC from *C. glutamicum*, which seems inhibited by acetyl-CoA, as well as ADP and AMP (Peters-Wendisch *et al*. 1997). Aspartate for microbial PCs and glutamate for vertebrate PCs can inhibit α_4_-type PCs via a mechanism distinct from that for acetyl-CoA, even though binding of aspartate is mutual exclusive with acetyl-CoA. The α_4_β_4_-type PCs are insensitive to aspartate as well. Inhibition by α-ketoglutarate, another dicarboxylate, seems to be more widely distributed, albeit not universal, as it can also modestly inhibit activity of α_4_β_4_-type PC from methanogenic archaea (Zeczycki, Maurice and Attwood 2010).

The only known post-translational modification of PC is its biotinylation, which is required for activity (Jitrapakdee and Wallace 1999). For mammals it was recently proposed that biotinylation of the five biotin-dependent carboxylases is a novel regulatory mechanism linked to the circadian clock (He *et al*. 2016). Evidence for a similar regulatory mechanism in bacteria does not exist. However, the efficiency of biotinylation is influenced by the allosteric effectors acetyl-CoA (positive) and aspartate (negative), acting on PC in *Geobacillus stearothermophilus* and *Saccharomyces cerevisiae* (Sundaram, Cazzulo and Kornberg 1971).

PC is present in many bacteria, but whereas for most eukaryotes it is the primary anaplerotic enzyme, in many bacteria it co-occurs with PEP carboxylase (PEPC) (Sauer and Eikmanns 2005). Notably, many enteric bacteria, including *E. coli*, lack PC and only seem to rely on PEPC for the production of oxaloacetate (Jitrapakdee *et al*. 2008). In non-photosynthetic eukaryotes, PC is also essential during gluconeogenesis, where the formed oxaloacetate is converted to PEP by PEP carboxykinase. Plants are also known to possess PC, where it might contribute to gluconeogenesis during germination (Jitrapakdee and Wallace 1999).

### Oxaloacetate decarboxylase (EC 4.1.1.112)

$$\begin{equation*}
{\rm{Oxaloacetate}} \leftrightarrow {\rm{Pyruvate}} + {\rm{C}}{{\rm{O}}_2}\quad {\Delta _{\rm{r}}}{G^{\prime {\rm{m}}}} = - 34.4 \pm 6.2{\rm{ kJ}}/{\rm{mol}}
\end{equation*}$$

Oxaloacetate decarboxylases (OAD) are enzymes that catalyze the irreversible decarboxylation of oxaloacetate to pyruvate. Many enzymes, including malic enzymes, pyruvate kinases, malate dehydrogenases, pyruvate carboxylases and PEP carboxykinases are known to have OAD ‘side-activity’. However, at least four different types of dedicated OADs have been described. We will try to give a concise summary of what is known about these different types, which deserve their own review.

Best known are the Na^+^-pumping, biotin-dependent OAD and the soluble, divalent cation-dependent OAD. These are the two classes of OADs that are typically acknowledged, and will be described in more detail below. However, in *Lactococcus lactis* and *Enterococcus faecalis* malic enzymes have been reported that have lost their ability to decarboxylate malate to pyruvate, and as such can be seen as dedicated OADs (Sender *et al*. 2004; Espariz *et al*. 2011). Mg^2+^ or Mn^2+^ are required for activity. Fumarate, succinate and NAD(P)H were found to inhibit their OAD activity, while these are positive effectors for many malic enzymes. The ME-related OAD takes part in the citrate fermentation pathway, converting the oxaloacetate formed by citrate lyase (Pudlik and Lolkema 2011). Additionally, a novel OAD from *Pseudomonas aeruginosa* has been characterized that belongs to the PEP mutase/isocitrate lyase superfamily (Narayanan *et al*. 2007). The enzyme is homotetrameric, and forms a complex with Mg^2+^ and oxalate. Orthologs are found in all Pseudomonad genomes, and a few other Gram-negative bacteria. Gene disruption showed that the enzyme is not essential, and the genomic context led the authors to speculate that the OAD helps in the supply of pyruvate or CO_2_ for a variety of cellular processes (Narayanan *et al*. 2007).

The Na^+^-pumping OADs belong to the biotin-dependent enzyme family, like the above described pyruvate carboxylase. Other known biotin-dependent Na^+^-pumping decarboxylases are methylmalonyl-CoA decarboxylase, glutaconyl-CoA decarboxylase, and malonate decarboxylase. In the biotin-dependent decarboxylases the carboxyl moiety bound to biotin is not transferred to a substrate. Instead, the CO_2_ is released at the membrane bound β-subunit in a reaction that consumes a periplasmic proton and is coupled to the translocation of up to two Na^+^ ions from the cytoplasm to the periplasm (Dimroth, Jockel and Schmid 2001; Lietzan and St. Maurice 2014). The Na^+^ gradient is then used to drive ATP-synthesis, solute uptake, or movement of flagella.

Na^+^-pumping decarboxylases consist of three different subunits: α, β and γ, of which the latter two are situated in the membrane. The quaternary structure of OAD has for long remained elusive, but for *Vibrio cholerae* the complex has now been shown to contain the α, β and γ subunits in a 4:2:2 ratio (Balsera, Buey and Li 2011; Inoue and Li 2015). More recently, the β and γ subunits of *S .typhimurium* OAD were found to form a β_3_γ_3_ hetero-hexamer, which likely binds up to 6 α subunits through a more dynamic association (Xu *et al*. 2020). Both the α and the γ subunits contain a Zn^+^ ion (Studer *et al*. 2007). The Na^+^-pumping OAD is found in both bacteria and archaea, but occurs mainly in proteobacteria. It is known to be important for citrate/tartrate fermentation, as are the other biotin-dependent Na^+^-pumping decarboxylases for the fermentation of other dicarboxylic acids (Dimroth and Schink 1998; Buckel 2001). Whether the Na^+^-pumping OAD is exclusively used in citrate/tartrate fermenting bacteria is not clear. To our understanding, there seems to be no clear reason why it should not be able to take part in other types of energy metabolism. At least one archaeal Na^+^-pumping OAD was (partly) characterized, from *Archaeoglobus fulgidus*, which does not ferment citrate, suggesting indeed that the role of Na^+^-pumping OADs is not restricted to citrate fermentation (Dahinden *et al*. 2004). In *E. faecalis* a variant of the Na^+^-pumping OAD had been described that has an additional subunit that allows activity in a soluble—non membrane associated—form (Repizo *et al*. 2013).

The soluble divalent cation-dependant OAD had been found and characterized in a range of different microorganisms already, until in 2010 the encoding gene was finally identified in *C. glutamicum* (Klaffl and Eikmanns 2010). The soluble OAD from *C. glutamicum* is reported to be homotetrameric as well as homodimeric (Jetten and Sinskey 1995; Klaffl and Eikmanns 2010), whereas the one from *Pseudomonas stutzeri* is monomeric (Labrou and Clonis 1999). Activity was absolutely dependent on Mn^2+^ or Mg^2+^, NAD^+^ was found to stimulate activity, and acetate and various dicarboxylic acids were found to act as inhibitors. ADP and ATP are both reported as inhibitors and activators (Benziman *et al*. 1978; Ng, Wong and Hamilton 1982; Jetten and Sinskey 1995; Labrou and Clonis 1999; Klaffl and Eikmanns 2010). The function of the soluble OAD is not quite clear. *C. glutamicum* did not appear to be affected by the deletion of the soluble OAD. Based on its relatively high K_m_ value for oxaloacetate, typically present in low concentrations, the authors suggested that the soluble OAD might function as an overflow mechanism (Klaffl and Eikmanns 2010). It has also been suggested that it might be required for the supply of pyruvate during growth on TCA-cycle intermediates (Benziman *et al*. 1978). Recently, a homologous OAD was identified in eukaryotes as well, potentially involved in maintaining an optimal oxaloacetate pool (Pircher *et al*. 2015; Etemad *et al*. 2019).

### Malate dehydrogenase (EC 1.1.1.37) (EC 1.1.1.82)

$$\begin{eqnarray*}
{\rm{Malate}} + {\rm{NA}}{{\rm{D}}^ {+} } \leftrightarrow {\rm{Oxaloacetate}} + {\rm{NADH}}\nonumber \\
\quad {\Delta _{\rm{r}}}{{\rm{G^{\prime}}}^{\rm{m}}} = 30.3 \pm 0.6{\rm{ kJ}}/{\rm{mol}}
\end{eqnarray*}$$

$$\begin{eqnarray*}
{\rm{Malate}} + {\rm{NAD}}{{\rm{P}}^ {+} } \leftrightarrow {\rm{Oxaloacetate}} + {\rm{NADPH}}\nonumber \\
\quad {\Delta _{\rm{r}}}{{\rm{G^{\prime}}}^{\rm{m}}} = 31.3 \pm 0.9{\rm{ kJ}}/{\rm{mol}}
\end{eqnarray*}$$

Malate dehydrogenase (MDH) catalyzes the reversible oxidation of malate to oxaloacetate using NAD(P)^+^. Another malate dehydrogenase exists (in bacteria) that uses a quinone instead of NAD(P)^+^, called malate:quinone oxidoreductase (MQO), which will be reviewed further down. The MDH reaction mechanism proceeds via the binding of NAD(P)^+^ followed by malate in a hydrophobic pocket, which is then closed by an external loop through a conformational change (Goward and Nicholls 1994; Minárik *et al*. 2002). The mechanism is identical to that of L-lactate dehydrogenase (LDH), which shares an evolutionary origin with MDHs.

MDHs are ubiquitously present in all domains of life. Two distinctive classes of MDHs exist: dimeric and tetrameric (Madern 2002). The latter is more closely related to LDHs, which also form tetramers, and occurs predominantly in prokaryotes; primarily in Gram-positives, while in archaea they are the only MDH present (Takahashi-Íñiguez *et al*. 2016). In some databases they can be wrongly annotated as LDH, and a single amino acid substitution has shown to convert an *E. coli* MDH to LDH. The dimeric MDHs are further split into two distinct phylogenetic groups. Both are present only in bacteria—in particular Gram-negatives (Takahashi-Íñiguez *et al*. 2016), and eukaryotes. In eukaryotes, this split follows mitochondrial (and glycosomal) MDHs versus cytosolic (and chloroplastic) MDHs (Madern 2002). In eukaryotes all MDHs are NAD^+^-dependent, except for the chloroplast MDHs, which use NADP^+^ (Minárik *et al*. 2002). Bacterial MDHs are predominantly NAD^+^-dependent; archaeal MDHs can be either NAD^+^- or NADP^+^-dependent, or have equal affinities for both (Takahashi-Íñiguez *et al*. 2016).

The precise dependency on the oligomeric state of MDHs is not known, but a study with monomeric mutants of the (dimeric) *E. coli* MDH showed a dramatic reduction in activity (Breiter, Resnik and Banaszak 1994), suggesting that at least the dimeric state is essential for activity. MDHs do not depend on any metal ions for activity, but are subject to some allosteric regulation, especially oxaloacetate seems to act as an inhibitor across the full spectrum of MDHs (Takahashi-Íñiguez *et al*. 2016). Mitochondrial MDHs are also activated by citrate in the oxaloacetate forming direction, while the reverse reaction is inhibited by citrate (Mullinax *et al*. 1982). NADP^+^-dependent MDHs of chloroplasts are activated by light, through the reduction of specific disulfide-residues. NADP^+^ inhibits the kinetics of this light-dependent activation (Scheibe and Jacquot 1983; Kagawa and Bruno 1988). Furthermore, MDHs both from mitochondria and *B. subtilis* (i.e. tetrameric) have been shown to form multi-enzyme complexes, or metabolons with other TCA-cycle enzymes, thereby regulating their activity and channelling the metabolites (Fahien *et al*. 1988; Meyer *et al*. 2011; Bartholomae *et al*. 2014; Jung and Mack 2018).

**Figure 6.**
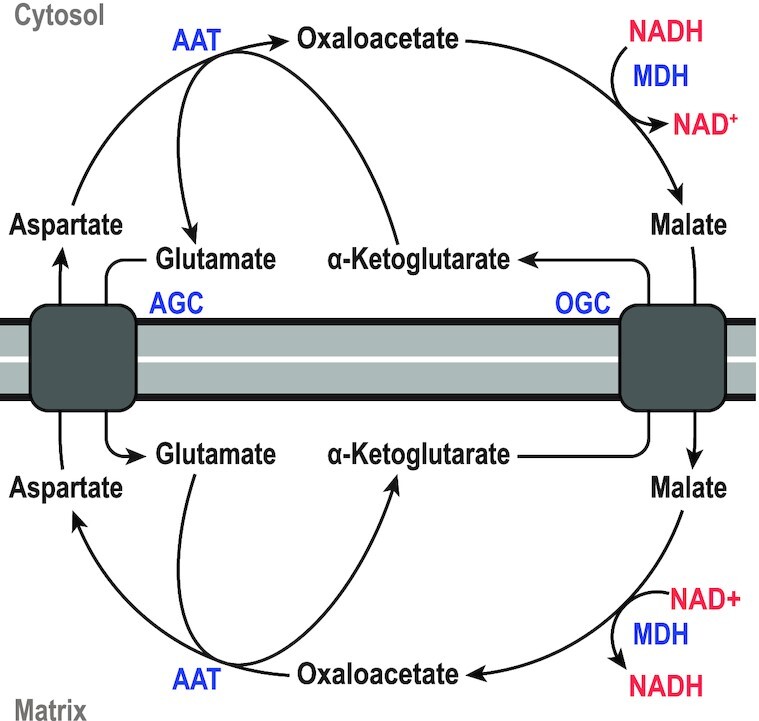
**The malate-aspartate shuttle**. The shuttle results in the net exchange of NADH with NAD^+^ from the cytosol into the mitochondrial matrix. AAT, aspartate aminotransferase; AGC, glutamate-aspartate antiporter; MDH, malate dehydrogenase; OGC, malate-α-ketoglutarate antiporter. Figure adapted from (Korla, Vadlakonda and Mitra 2015).

MDHs primarily function in the TCA-cycle, converting malate to oxaloacetate; generating NADH that is subsequently used by the respiratory complex. Additionally in eukaryotes, MDH—both the mitochondrial and the cytosolic isoforms, together with aspartate aminotransferase, the malate-α-ketoglutarate antiporter, and the glutamate-aspartate antiporter are responsible for the transport of electrons (i.e. NADH from glycolysis) from the cytosol into the mitochondrial matrix, to be used for oxidative phosphorylation (Lu *et al*. 2008). This is known as the malate-aspartate shuttle, which is depicted in Fig. 6. Furthermore, MDH is involved in the fermentation pathway to succinic acid via the reductive branch of the TCA-cycle (Thakker *et al*. 2012), and it has been shown to be involved in the formation of pyruvate from PEP via the so-called ‘malate shunt’. In this shunt, PEP is converted to pyruvate via PEPCK, MDH, and then ME—all part of the PPO-node. The primary function of this shunt is likely the trans-hydrogenation of NADH (oxidized by MDH) to NADPH (reduced by ME) (McKinlay *et al*. 2007; Taillefer *et al*. 2015; Olson *et al*. 2016).

### Malate:quinone oxidoreductase (EC 1.1.5.4)

$$\begin{eqnarray*}
{\rm{Malate}} + {\rm{Ubiquinone}} \leftrightarrow {\rm{Oxaloacetate}} + {\rm{Ubiquinol}}\nonumber \\
\quad {\Delta _{\rm{r}}}{{\rm{G^{\prime}}}^{\rm{m}}} = - 54.4 \pm 5.9\;{\rm{kJ}}/{\rm{mol}}
\end{eqnarray*}$$

$$\begin{eqnarray*}
{\rm{Malate}} + {\rm{Menaquinone}} \leftrightarrow {\rm{Oxaloacetate}} + {\rm{Menaquinol}}\nonumber \\
\quad {\Delta _{\rm{r}}}{{\rm{G^{\prime}}}^{\rm{m}}} = - 18.3 \pm 5.9\;{\rm{kJ}}/{\rm{mol}}
\end{eqnarray*}$$

Just like MDH, malate:quinone oxidoreductase (MQO) facilitates the oxidation of malate to oxaloacetate, but uses quinone, rather than NAD(P)^+^, rendering the reaction physiologically irreversible. The enzyme is membrane associated and contains a tightly non-covalently bound prosthetic FAD-group (Molenaar, van der Rest and Petrovic 1998). However, no crystal structure is available at the moment, and details about the precise reaction mechanism are lacking.

MQOs are present mostly in bacteria, with only a handful of archaeal MQOs known, but none characterized. One study found MQOs to be present in 27% of analysed bacterial genomes. Eukaryotic MQOs are almost exclusively limited to Apicomplexa, a large phylum of parasitic protists (Mogi *et al*. 2009; Kabashima *et al*. 2013; Marreiros *et al*. 2016). MQOs can be split in two clear phylogenetic groups, with the second—minor—group being restricted mainly to Apicomplexa, Archaea and ϵ-proteobacteria (Mogi *et al*. 2009). Some Apicomplexa, Archaea and ϵ-proteobacteria are also known to possess MQOs from the major group, however. It is not clear whether there is a functional difference between the two phylogenetic groups.

Both ubiquinone and menaquinone can function as electron acceptor, however, many bacteria only possess menaquinone, which has a much lower redox potential (−74 mV versus + 113 mV) and therefore potentially allows the (reverse) reduction of oxaloacetate (Kather *et al*. 2000). Nevertheless, this is still much higher than that of NAD^+^/NADH (−320 mV) (Molenaar, van der Rest and Petrovic 1998). When both MDH and MQO are present in the same organism—as is very often the case, the simultaneous oxidation of malate (by MDH) and reduction of oxaloacetate (by MQO) can take place. This effectively channels electrons from NADH to quinone, mimicking the reaction of the non-proton pumping type II NADH dehydrogenase (NDH2), an enzyme believed to share a common ancestor with MQO (Mogi *et al*. 2009). In fact, there is evidence that in *C. glutamicum*, this is indeed one of several mechanisms for NADH:quinone oxidoreductase activity, and in *Mycobacterium smegmatis*, NDH2 mutants could be complemented by MDH (Miesel *et al*. 1998; Nantapong *et al*. 2004; Cook *et al*. 2014).

In organisms that contain MQO, but lack MDH, such as *Helicobacter pylori*, it clearly functions as part of the TCA-cycle (Kather *et al*. 2000). In *Staphylococcus aureus* MQO is also important for resistance against nitric oxide—a weapon of the host immune system—and the ensuing virulence (Spahich *et al*. 2016). In *P. aeruginosa* and *Pseudomonas citronellolis*, MQO was found to be essential for growth on acetate and ethanol, through the glyoxylate cycle, but nor for growth on glucose, lactate, succinate or malate (Görisch *et al*. 2002; Förster-Fromme and Jendrossek 2005). What the precise role of MQO is in organisms that have both MQO and MDH is not fully understood. It could be the observed NADH:quinone oxidoreductase activity as discussed above. Further observations are that in *C. glutamicum* MQO was the main contributor to the TCA-cycle, compared to MDH (Molenaar *et al*. 2000), while in *E. coli*, to opposite was true (van der Rest, Frank and Molenaar 2000).

### Malic enzyme (EC 1.1.1.38) (EC 1.1.1.39) (EC 1.1.1.40)

$$\begin{eqnarray*}
{\rm{Malate}} + {\rm{NA}}{{\rm{D}}^ {+} } \leftrightarrow {\rm{Pyruvate}} + {\rm{C}}{{\rm{O}}_2} + {\rm{NADH}}\nonumber \\
\quad {\Delta _{\rm{r}}}{{\rm{G^{\prime}}}^{\rm{m}}} = - 4.1 \pm 6.2{\rm{ kJ}}/{\rm{mol}}
\end{eqnarray*}$$

$$\begin{eqnarray*}
{\rm{Malate}} + {\rm{NAD}}{{\rm{P}}^ {+} } \leftrightarrow {\rm{Pyruvate}} + {\rm{C}}{{\rm{O}}_2} + {\rm{NADPH}}\nonumber \\
\quad {\Delta _{\rm{r}}}{{\rm{G^{\prime}}}^{\rm{m}}} = - 3.1 \pm 6.2{\rm{ kJ}}/{\rm{mol}}
\end{eqnarray*}$$

Malic enzymes (ME) catalyze the reversible decarboxylation reaction from malate to pyruvate via the reduction of NADP^+^ or NAD^+^, proceeding in three steps: First, the dehydrogenation of malate to oxaloacetate, followed by the decarboxylation where enolpyruvate is formed, which is converted to pyruvate via the tautomerization reaction. The catalysis requires a divalent cation bound in the active site (Tao, Yang and Tong 2003). ME variants are classified in various ways. Typically, they are classified based on their cofactor usage (NADP^+^ versus NAD^+^) and the ability to decarboxylate oxaloacetate (OAA), in the following three classes: EC1.1.1.38 (NAD^+^-dependent; OAA decarboxylating), EC1.1.1.39 (NAD^+^-dependent) and EC 1.1.1.40 (NADP^+^-dependent; OAA decarboxylating). However, NADP^+^-dependent MEs that do not decarboxylate OAA are also described, but they do not have their own EC-number (Fukuda *et al*. 2005; Taillefer *et al*. 2015; Olson *et al*. 2016).

Phylogenetically, there are two main groups. The first group is exclusively prokaryotic, with the exception of *Trichomonas vaginalis* and *E. histolytica*, and has subunits of about 40–50 kDa, which is smaller than group 2 ME subunits of about 60 kDa. Group 2 MEs are often referred to as the ‘eukaryotic’ MEs, however, they also occur in prokaryotes. NAD^+^ or NADP^+^ dependency does not appear to correlate very strongly with the phylogeny (Tronconi, Andreo and Drincovich 2018), explained from the fact that only minor amino acid changes are needed to change the specificity (Hsieh, Chen and Hung 2011). Whether or not OAA decarboxylation correlates better with phylogeny is less clear; the EC1.1.1.39 (NAD^+^-dependent; non-OAA decarboxylating) ME class is exclusively composed of plant mitochondrial MEs. However, the *C. thermocellum* and *T. kodakarensis* MEs, shown to be non-decarboxylating NADP^+^-MEs belongs to the group 1 MEs (Fukuda *et al*. 2005; Taillefer *et al*. 2015). Group 2 MEs can subsequently also be divided in two clades, but these—recently reviewed—details are beyond the scope of this review (Doležal *et al*. 2004). Group 2 also include the malolactic enzyme from lactic-acid bacteria that catalyzes the reversible decarboxylation reaction from malate to lactate via the reduction of NAD^+^, and the oxaloacetate decarboxylases described earlier (Tronconi, Andreo and Drincovich 2018).

The group 2 MEs are typically active as homotetramers, with the exception of the plant mitochondrial MEs (EC1.1.1.39), which form heterodimers of two ME paralogs. However, reports of homodimeric, -hexameric, -octameric and -decameric forms are also known (Suye *et al*. 1992; Tronconi *et al*. 2008). Group I MEs are also primarily found as tetramers (Kobayashi *et al*. 1989; Chen *et al*. 1998). The two characterized archaeal MEs from *Sulfolobus solfataricus* and *T. kodakarensis*, however, are both dimeric (Bartolucci *et al*. 1987; Fukuda *et al*. 2005), as well as the ones from the eukaryotes *T. vaginalis* and *E. histolytica*, the latter of which is also closely related to the archaeal MEs (Doležal *et al*. 2004). Many type I MEs are described that have a molecular mass of around 80–90 kDa, due to a fused C-terminal domain homologous to phosphate acetyltransferase (PTA). The PTA domain is not essential for ME activity, nor does it show any PTA activity (Mitsch *et al*. 1998). Instead, it was found that deletion of the PTA domain from the *E. coli* enzyme abolished allosteric inhibition by acetyl-CoA and activation by glutamate, aspartate, and glucose-6-P, indicating that the PTA domain is important for allosteric control (Bologna, Andreo and Drincovich 2007). *Rhizobium meliloti*, a Gram-negative nitrogen fixing bacterium contains two group 1 MEs, both with PTA domains, with divergent allosteric regulation, of which only one is sensitive to acetyl-CoA (Voegele, Mitsch and Finan 1999). *E. coli* contains ME isoforms of both groups. Both are inhibited by fumarate and oxaloacetate, and activated by aspartate. Interestingly, acetyl-phosphate inhibits the group 2 ME while it activates the group 1 ME, independently of the PTA domain (Bologna, Andreo and Drincovich 2007). Many other allosteric regulators for MEs are known, including NH_4_^+^ and ATP (Kawai *et al*. 1996; Hsieh, Chen and Hung 2011). The complex allosteric regulation, often varying between paralogs, illustrates the different roles that can be played by ME and PPO-node enzymes in general.

Generally, NAD^+^-dependent MEs are used in the catabolism of malate, and NADP^+^-dependent MEs are used for NADPH generation(Sauer and Eikmanns 2005). Although both have been shown to be required for acetyl-CoA generation via pyruvate, primarily NADP^+^-dependent MEs are thought to be responsible for gluconeogenesis (Voegele, Mitsch and Finan 1999; Sauer and Eikmanns 2005; Bologna, Andreo and Drincovich 2007). Only one example is known of a wildtype bacterium that uses a NADP^+^-dependent ME to fix CO_2_ (Matula, McDonald and Martin 1969).

### Phosphotransferase system

$$\begin{eqnarray*}
{\rm{Glucos}}{{\rm{e}}_{{\rm{ex}}}} + {\rm{Phosphoenolpyruvate}} \leftrightarrow {\rm{Glucose}} - 6 - {{\rm{P}}_{{\rm{in}}}} \nonumber \\
\qquad + {\rm{pyruvate}}\nonumber \\
\quad {\Delta _{\rm{r}}}{{\rm{G^{\prime}}}^{\rm{m}}} - 44.9 \pm 1.2 = {\rm{kJ}}/{\rm{mol}}
\end{eqnarray*}$$

**Figure 7.**
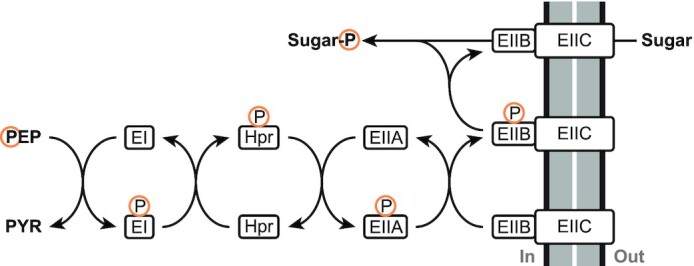
Sequence of reactions in the phosphotransferase system.

The phosphotransferase system (PTS) is a system consisting of several proteins that is involved in active uptake of sugars, driven by the transfer of the phosphate group from PEP to the imported sugar. As such, it is simultaneously responsible for sugar phosphorylation and the conversion of PEP to pyruvate. The mechanism proceeds as follows: First, the phosphate group of PEP is transferred to Enzyme I (EI), after which the phosphate group is transferred sequentially from EI to histidine protein (HPr), then to Enzyme II A (EIIA), and finally to Enzyme II B (EIIB). EIIB then phosphorylates the sugar as it is imported through the cell membrane via the transmembrane Enzyme II C (EIIC). Each of these steps is reversible (Deutscher, Francke and Postma 2006). The complete sequence of steps is shown in Fig. 7. The sugar phosphorylation increases the polarity of the sugar, decreasing the rate at which it can leak out of the cell again (Bar-Even *et al*. 2012), while simultaneously keeping a low intracellular concentration of the non-phosphorylated sugar, maintaining a high driving force. The EII proteins are specific for the sugar they transport, and many different EII proteins can be present in parallel in one cell that are all connected to a common PEP/EI/HPr pathway. For example, *E. coli* contains at least 15 different EII complexes (Deutscher, Francke and Postma 2006).

The PTS is almost exclusively present in bacteria, and only recently it has been identified and characterized in archaea (Pickl, Johnsen and Schonheit 2012; Cai *et al*. 2014); the PTS proteins are completely absent from eukaryotes. A comparative genomics analysis in 2005 found that 57% of studied bacteria possessed genes for all required PTS constituents, while 22% did not possess any (Barabote and Saier 2005). The remaining 21% contained soluble ‘PTS’ proteins, which are also known to possess regulatory functions, which will be discussed briefly below. Complete systems have been found in almost all bacterial kingdoms, and have been gained and lost with high frequency.

Seven different PTS types are distinguished, of which the substrate-recognizing EIIC proteins have evolved from at least four independent sources, giving rise to the Glc-Fru-Lac superfamily, the Asc-Gat superfamily, the Man family and the Dha family. Overall, the different EII components form a true mosaic, derived from many ancestral sources with a complex phylogeny between the different PTS types. EI exhibits both sequence and structural similarity with PPdK (Oberholzer *et al*. 2005; Deutscher, Francke and Postma 2006). EIIA, B and C can either exist as separate proteins, or may be fused in various combinations (Barabote and Saier 2005). The individual components of the PTS each form functional dimers (or oligomers), including the transmembrane EIIC, but there is no evidence for the existence of larger complexes or metabolons between EI, HPr and EII (Deutscher, Francke and Postma 2006; Patel *et al*. 2006; McCoy, Levin and Zhou 2015).

As mentioned before, the PTS also possesses regulatory functions, which are related to carbon, nitrogen and phosphate utilizations, as well as virulence in some pathogens, chemotaxis and potassium transport (Galinier and Deutscher 2017). By and large, the signal for these processes is provided by the phosphorylation state of the PTS components, which is dictated by the metabolic state of the cell, and the availability of its substrates (Deutscher *et al*. 2014). The signal is transduced via phosphorylation of non-PTS proteins by PTS components or via protein-protein interactions. In enteric bacteria, EIIA of the glucose PTS plays a central role in regulating carbon metabolism, in particular for catabolite repression and the uptake of sugars (Somavanshi, Ghosh and Sourjik 2016), making it a common target for metabolic engineering.

Although PTSs are only known to facilitate active solute import and not the export, EI was found recently to facilitate a significant (gluconeogenic) reverse flux from pyruvate to PEP in *E. coli*—both in wild type and in a PPS knock-out mutant during gluconeogenic as well as glycolytic growth (Long *et al*. 2017). The responsible phosphate donor is not known, but it suggests that the PTS might have a more central metabolic role (in the PPO-node) beyond sugar uptake.

## PHYLOGENETIC DISTRIBUTION OF PPO-NODE ENZYMES



**Figure 8.**
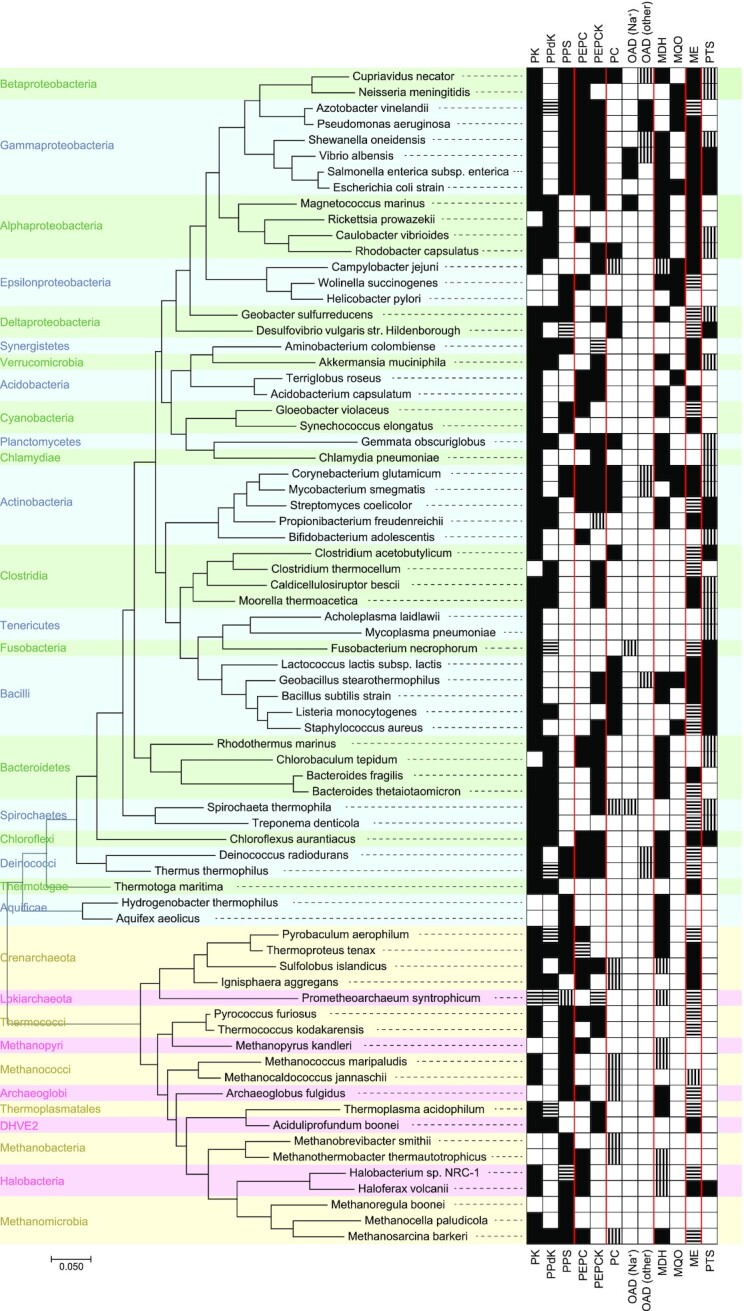
**Different subsets of PPO-node enzymes as present in selected (cultivated) prokaryotes, spread-out through the tree of life**. Presence of an enzyme is based on data from the UniProt database, with vertical stripes indicating presence of a corresponding enzyme commission (EC) number in the given organism, and horizontal stripes the presence of corresponding InterPro accession numbers; fully black indicates the presence of both. The results are a conservative estimation, as not all enzymes have (unique) InterPro accession numbers (i.e. a described protein families) or annotated EC numbers. The PPi-dependent PEPCK, the soluble OAD and the ME-related OAD do not have InterPro protein families described for them. Conversely, for multi-subunit proteins (i.e. Na^+^-pumping OAD, and PTS-systems) the EC numbers are the same for the different subunits, and can therefore yield an overestimation. Finally, some MDHs are known to be annotated as LDHs, and might also not be picked up (Taillefer *et al*. 2015). The phylogenetic tree is the result of the Maximum Likelihood algorithm, based on 16s rRNA sequences that were aligned using MUSCLE. A list of the selected EC numbers and InterPro accession numbers can be found in the supplementary data.

No single organism harbours enzymes to catalyse all 11 reactions described above. Instead, they have different subsets of those, illustrated by the global overview presented in Fig. 8. From the overview it is already evident that most of the PPO-node enzymes are distributed over both Bacteria and Archaea. Although OADs, MQOs and PTSs are rare in Archaea. And with the exception of the PTS, Eukaryotes—absent from the figure—also possess enzymes for all of the PPO-node reactions; albeit occurrence of PPS and MQO is very restricted. The ubiquity and especially the pervasiveness of many of the PPO-node enzymes amongst prokaryotes suggest that they are evolutionary old and that some may have been present already in the last universal common ancestor, LUCA. Conversely, the existence of multiple enzymes/routes connecting PEP, pyruvate and oxaloacetate (as well as malate) could also be indicative of convergent evolution, in which case they emerged in parallel after LUCA (and were then distributed through horizontal gene transfer, to account for their pervasiveness). The study into which genes were present in LUCA is still a contentious, but mostly nebulous subject. A phylogenetic study from 2016 nevertheless gives the first small insight into LUCA's physiology and habitat (Weiss *et al*. 2016). LUCA's metabolism likely relied on H_2_ dependent CO_2_ fixation through the Wood-Ljungdahl pathway, which would indicate that the PPO-node primarily had a gluconeogenic role, to generate the precursor metabolites upwards from acetyl-CoA (Fig. 1). What the composition of that PPO-node would have been—if even a real node—is still an open question.

Perhaps the most striking from Fig. 8 is how limited the correlation seems to be between the organisms’ phylogeny and the PPO node composition, despite its place at the heart of the core metabolism (Fig. 1). It is illustrative of the evolutionary flexibility of metabolism in general and suggests that the PPO-node composition is strongly dictated by the organism's niche, together with its evolutionary history.

## CONCLUDING REMARKS

Many of the PPO-node enzymes are often referred to as either glycolytic, gluconeogenic, anaplerotic or cataplerotic. Although those are typical roles they can fulfil, such generalizations do not do justice to the varying and dynamic roles certain PPO-node enzymes play in different metabolisms, of which several examples have been discussed in this review. The most extreme example in this regard is PPS, which—thermodynamically—highly favours the gluconeogenic conversion of pyruvate to PEP, and yet has been shown to function as a glycolytic enzyme in several Archaea, despite the simultaneous presence of a PK in the genome, and of course the existence of PPdK, which is even more ambivalent with respect to its role in glycolysis and gluconeogenesis. Similarly, the role of PEPCK can be either glycolytic, gluconeogenic, anaplerotic, or cataplerotic. Also, the quintessentially-glycolytic PK is required for growth on gluconeogenic substrates in certain cases. And finally, their variability and dynamicity are further exemplified by the complex allosteric control that many PPO-node enzymes are subjected to, which often vary (or even work oppositely) for different homologs and paralogs.

So, not only can PPO-node enzymes have different roles in different organisms, but also within one organism can they operate in opposing directions, or seemingly contradicting roles. We therefore recommend that using the labels *glycolytic*, *gluconeogenic*, *anaplerotic* and *cataplerotic* to define PPO-node enzymes should be avoided, as it might well only be a partial description; but also to prevent being put on the wrong track while trying to understand an organism's metabolism.

## Supplementary Material

fuaa061_Supplemental_File

## Footnotes

1 Two pathways exist for *de novo* lysine biosynthesis: the diaminopimelate (DAP) pathway and α–aminoadipate (AAA) pathway. Only the former uses pyruvate as precursor, and is found in prokaryotes and plants. The AAA pathway occurs primarily in yeasts and fungi, but some hyperthermophilic prokaryotes also possess a variant of the AAA pathway (Bowman *et al*. 1988).

2 The only two variations known—both occurring in Archaea—are glyceraldehyde-3-phosphate:ferredoxin oxidoreductase (GAPOR) replacing glyceraldehyde-3-phosphate dehydrogenase (GAPDH), and the non-phosphorylating glyceraldehyde-3-phosphate dehydrogenase (GAPN), which also replaces phosphoglycerate kinase (PGK), abolishing the accompanying ATP generation (Brasen *et al*. 2014).

3 Pyruvate phosphate dikinase is normally written as pyruvate, phosphate dikinase, using a comma. However, to improve readability, we have written it without a comma throughout this review.

4 An example of such a different prioritization can be found in some extreme thermophiles, which do not phosphorylate certain glycolytic metabolites. At very high temperatures those become unstable and can undergo spontaneous dephosphorylation (Bar-Even *et al*. 2012; Brasen *et al*. 2014). The consequence is that non-phosphorylated compounds are more prone to membrane leakage (due to their increased hydrophobicity), but at higher temperature this constraint seemingly weighs less than loss in energy resulting from spontaneous dephosphorylation. Other alternative prioritizations could relate to the trade-off between rate and yield of energy conservation; toxicity of metabolic intermediates; the costs for the required enzyme machinery; requirement for metals, cofactors & coenzymes, or ecologically rare/limiting elements; etc.

5 Values for the Gibbs free energy of the reaction (ΔrG) are obtained from eQuilibrator (Flamholz *et al*. 2012). The ‘m’ in ΔrG′m indicates that in the calculations 1 mM was used as concentration for all the reactants, at a pH of 7.0 and ionic strength of 1.0 M. ΔrG is defined such that at negative values, the net reaction proceeds in the forward direction. However, it is good to keep in mind that ΔrG′m does not reflect the true ΔrG, which is ultimately determined by the actual concentrations of the reactants; it essentially reflects to which direction (and extend) the equilibrium leans. A good example is glyceraldehyde 3-phosphate dehydrogenase, which has a ΔrG′m of 25.9 kJ/mol in the glycolytic direction, yet it still proceeds (because the concentrations of the substrates are many times higher than that of the products). The often-large error of ΔrG′m is a result of the fact that for only ∼10% of the metabolic reactions in E. coli and human cell metabolic models, experimental equilibrium data are available and that for the remainder the group contribution method is used. This method can cover the majority (∼80%) of relevant biochemical reactions, but is less accurate since it relies on a simplifying assumption that the contributions of groups are additive. Thus, the average estimation error attributed to group contribution is about 9–10 kJ/mol (Noor *et al*. 2013).
