# Supplementary material for: The PEP-pyruvate-oxaloacetate node: variation at the heart of metabolism
Source: FEMS Microbiol Rev. 2020 Dec 8;45(3):fuaa061. doi: 10.1093/femsre/fuaa061 (PMC8100219; doi:10.1093/femsre/fuaa061)
Supplement: fuaa061_Supplemental_File [file fuaa061_supplemental_file.docx]

**Supplementary data for Figure 8**

To compose Figure 8, a full list of entries (including the associated taxon identifiers) was downloaded from the UniProt database (<https://www.uniprot.org/>) for each of the selected InterPro protein families and E.C. numbers (Supplementary table 1). Each of those lists was then used to assess whether the selected organisms’ taxon identifiers (Supplementary table 2) were present or not, in order to confer presence (or absence) of the different PPO-node enzymes in those organisms.

In order for OAD (Na+) presence to be conferred based on InterPro protein families, alpha, beta, and gamma subunits were required to be present simultaneously.

In order for PTS presence to be conferred based on InterPro protein families, EI, HPr, IIA, IIB, and IIC subunits were required to be present simultaneously; IID was ignored.

Many enzymes are divided in multiple InterPro protein families; only one was required to be present per enzyme/subunit, in order for it to be conferred as present. Conversely, not all described enzymes belong to a (unique) InterPro protein family (i.e. PEPCK (PP), soluble OAD, ME-related OAD), and will therefore not be detected, resulting in an underestimation.

The different subunits of multimeric enzymes (i.e. OAD (Na+), MDH, and PTS) have the same EC number associated with it. In those cases, presence of the EC number can yield an overestimation if not all required subunits are actually present in the organism.

**Supplementary table 1**: List of InterPro protein families and EC numbers used to confer presence of PPO-node enzymes in selected organisms.

| **Enzyme** | **Subunits or subclasses** | **InterPro protein family** | **EC number** |
| --- | --- | --- | --- |
| PK |  | IPR001697 | 2.7.1.40 |
| PPdK |  | IPR010121 | 2.7.9.1 |
| PPS |  | IPR006319 | 2.7.9.2 |
| PEPC |  | IPR021135  IPR007566  IPR022805 | 4.1.1.31 |
| PEPCK (ATP) |  | IPR001272 | 4.1.1.49 |
| PEPCK (PTP) |  | IPR008209 | 4.1.1.32 |
| PEPCK (PP) |  | - | 4.1.1.38 |
| PC |  | IPR005930 | 6.4.1.1 |
| OAD (Na+) | Alpha | IPR005776 | 7.2.4.2 |
|  | Beta | IPR005661 |  |
|  | Gamma | IPR023424 |  |
| OAD (other) | Soluble | - | 4.1.1.112 |
|  | PEP mutase/isocitrate lyase superfamily | IPR023687 |  |
|  | ME-related OAD | - |  |
| MDH |  | IPR011275  IPR011273  IPR010097  IPR011274  IPR010945  IPR023958 | 1.1.1.37  1.1.1.82  1.1.1.299 |
| MQO |  | IPR006231 | 1.1.5.4 |
| ME |  | IPR001891  IPR012188  IPR023667 | 1.1.1.38  1.1.1.39  1.1.1.40  1.1.1.83 |
| PTS ** | EI | IPR024692 | 2.7.3.9 |
|  | HPr | IPR000032 |  |
|  | IIA | IPR036665  IPR036542  IPR036662 |  |
|  | IIB | IPR036095  IPR036878  IPR036667 |  |
|  | IIC | IPR013853  IPR004796  IPR004700  IPR004703  IPR004699 |  |
|  | IID | IPR004704 |  |

**Supplementary table 2:** List of selected organisms and corresponding organism identifiers used to find InterPro protein families and EC numbers, as well as accession numbers of 16s sequences used to construct the phylogenetic tree.

| **Organism** | **Fhylum/class** | **Taxon identifier** | **Accession number 16s rRNA gene** |
| --- | --- | --- | --- |
| Acidobacterium capsulatum | Acidobacteria | 240015 | NR_043386.1 |
| Terriglobus roseus | Acidobacteria | 926566 | NR_043918.1 |
| Bifidobacterium adolescentis | Actinobacteria | 367928 | NR_074802.2 |
| Corynebacterium glutamicum | Actinobacteria | 196627 | NR_041817.1 |
| Mycobacterium smegmatis | Actinobacteria | 246196 | AB305022.1 |
| Propionibacterium freudenreichii | Actinobacteria | 66712 | LN624749.1 |
| Streptomyces coelicolor | Actinobacteria | 100226 | NR_112305.1 |
| Caulobacter vibrioides | Alphaproteobacteria | 565050 | NR_037099.1 |
| Magnetococcus marinus | Alphaproteobacteria | 156889 | NR_074371.1 |
| Rhodobacter capsulatus | Alphaproteobacteria | 272942 | NR_043407.1 |
| Rickettsia prowazekii | Alphaproteobacteria | 272947 | NR_044656.2 |
| Aquifex aeolicus | Aquificae | 224324 | NR_114796.2 |
| Hydrogenobacter thermophilus | Aquificae | 608538 | NR_075004.2 |
| Bacteroides fragilis | Bacteroidetes | 272559 | NR_074784.2 |
| Bacteroides thetaiotaomicron | Bacteroidetes | 226186 | NR_074277.1 |
| Rhodothermus marinus | Bacteroidetes | 518766 | NR_029282.2 |
| Cupriavidus necator | Betaproteobacteria | 381666 | NR_028766.1 |
| Neisseria meningitidis | Betaproteobacteria | 122586 | NR_104946.1 |
| Akkermansia muciniphila | Verrucomicrobia | 349741 | NR_042817.1 |
| Chlamydia pneumoniae | Chlamydiae | 83558 | NR_026527.1 |
| Chlorobaculum tepidum | Chlorobi | 194439 | NR_044685.2 |
| Chloroflexus aurantiacus | Chloroflexi | 324602 | NR_043411.1 |
| Gloeobacter violaceus | Cyanobacteria | 251221 | NR_074282.1 |
| Synechococcus elongatus | Cyanobacteria | 1140 | NR_074309.1 |
| Deinococcus radiodurans | Deinococcus/Thermus | 243230 | NR_026401.1 |
| Thermus thermophilus | Deinococcus/Thermus | 300852 | NR_037066.1 |
| Desulfovibrio vulgaris | Deltaproteobacteria | 882 | NR_074446.1 |
| Geobacter sulfurreducens | Deltaproteobacteria | 243231 | NR_029179.1 |
| Campylobacter jejuni | Epsilonproteobacteria | 192222 | NR_041834.1 |
| Helicobacter pylori | Epsilonproteobacteria | 85962 | NR_044761.1 |
| Wolinella succinogenes | Epsilonproteobacteria | 273121 | NR_025942.1 |
| Bacillus subtilis | Firmicutes | 224308 | NR_112116.2 |
| Caldicellulosiruptor bescii | Firmicutes | 521460 | NR_074788.1 |
| Clostridium acetobutylicum | Firmicutes | 272562 | NR_074511.2 |
| Clostridium thermocellum | Firmicutes | 203119 | L09173.1 |
| Geobacillus stearothermophilus | Firmicutes | 1422 | NR_115284.2 |
| Lactococcus lactis | Firmicutes | 272623 | KC429785.1 |
| Listeria monocytogenes | Firmicutes | 169963 | NR_044823.1 |
| Moorella thermoacetica | Firmicutes | 264732 | NR_113196.1 |
| Staphylococcus aureus | Firmicutes | 196620 | NR_118997.2 |
| Fusobacterium necrophorum | Fusobacteria | 1441736 | LC011038.1 |
| Azotobacter vinelandii | Gammaproteobacteria | 322710 | MH048906.1 |
| Escherichia coli | Gammaproteobacteria | 83333 | NR_024570.1 |
| Pseudomonas aeruginosa | Gammaproteobacteria | 208964 | AF094715.1 |
| Salmonella typhimurium | Gammaproteobacteria | 588858 | NR_074910.1 |
| Shewanella oneidensis | Gammaproteobacteria | 211586 | NR_074798.1 |
| Vibrio cholerae | Gammaproteobacteria | 243277 | NR_044050.1 |
| Gemmata obscuriglobus | Planctomycetes | 114 | NR_037010.1 |
| Spirochaeta thermophila | Spirochaetes | 665571 | NR_074795.1 |
| Treponema denticola | Spirochaetes | 243275 | NR_074582.1 |
| Aminobacterium colombiense | Synergistetes | 572547 | NR_074624.1 |
| Acholeplasma laidlawii | Tenericutes | 441768 | NR_074448.2 |
| Mycoplasma pneumoniae | Tenericutes | 272634 | AF132741.1 |
| Thermotoga maritima | Thermotogae | 243274 | NR_102775.2 |
| Archaeoglobus fulgidus | Archaeoglobi | 224325 | NR_119237.1 |
| Ignisphaera aggregans | Crenarchaeota | 583356 | NR_043512.1 |
| Pyrobaculum aerophilum | Crenarchaeota | 178306 | NG_041958.1 |
| Sulfolobus islandicus | Crenarchaeota | 429572 | AY247901.1 |
| Thermoproteus tenax | Crenarchaeota | 768679 | NR_044683.1 |
| Aciduliprofundum boonei | DHVE2 | 439481 | DQ451875.1 |
| Halobacterium salinarum | Halobacteria | 64091 | AB603514.1 |
| Haloferax volcanii | Halobacteria | 309800 | NR_113448.1 |
| Methanobrevibacter smithii | Methanobacteria | 420247 | NR_044786.1 |
| Methanothermobacter thermautotrophicus | Methanobacteria | 187420 | NR_074260.1 |
| Methanocella paludicola | Methanocellales | 304371 | NR_074192.1 |
| Methanocaldococcus jannaschii | Methanococci | 243232 | NR_074233.1 |
| Methanococcus maripaludis | Methanococci | 267377 | NR_104984.1 |
| Methanoregula boonei | Methanomicrobiales | 456442 | NR_074180.1 |
| Methanopyrus kandleri | Methanopyri | 190192 | NR_074539.1 |
| Methanosarcina barkeri | Methanosarcinales | 269797 | NR_025303.1 |
| Pyrococcus furiosus | Thermococci | 186497 | AB603518.1 |
| Thermococcus kodakarensis | Thermococci | 69014 | NR_028216.1 |
| Thermoplasma acidophilum | Thermoplasmatales | 273075 | NR_028235.1 |
